# Total life expectancy and disability-free life expectancy and differences attributable to cigarettes’ smoking among Chinese middle-aged and older adults

**DOI:** 10.1186/s12877-024-05007-z

**Published:** 2024-08-08

**Authors:** Guogui Huang, Yao Pan, Yanan Luo

**Affiliations:** 1 Centre for Health Systems and Safety Research, Australia Institute of Health and Innovation, Faculty of Medicine, Health and Human Sciences, Macquarie University, Sydney, Australia; 2 Centre for Workforce Futures, Macquarie Business School, Macquarie University, Sydney, Australia; 3 School of Economics, Zhongnan University of Economics and Law, Wuhan, China; 4 Department of Global Health, School of Public Health, Peking University, Beijing, China; 5 Institute for Global Health and Development, Peking University, Beijing, China; 6 Department of Global Health, School of Public Health, No. 38 Xueyuan Road, Haidian District, Beijing, 100191 P.R. China

**Keywords:** Smoking, Disability-free life expectancy, Middle-aged and older adults, China

## Abstract

**Objectives:**

Middle-aged and older adults smoking for years are afflicted by smoking-related diseases and functional limitations; however, little is known about the effect of smoking on nonfatal conditions in middle and later life. This study aims to investigate the impact of smoking on both total life expectancy (TLE) and disability-free life expectancy (DFLE) and the variations in such effects by educational level in China.

**Methods:**

Data were drawn from the China Health and Retirement Longitudinal Study (CHARLS), 2011–2018, with a total sample of 16,859 individuals aged 45 years or older involved in the final analysis. The Activities of Daily Living (ADL) scale was used to measure disability, and the population-based multistate life table method was used to estimate the differences in TLE and DFLE by smoking status and educational attainment.

**Results:**

At baseline, 28.9% of participants were current smokers, 8.5% were former smokers, and 62.6% never smoked. Approximately 5.6% were identified with ADL disability. Both current smokers and former smokers experienced lower TLE and DFLE than never smokers, and such differences were particularly prominent among men. Intriguingly, former smokers manifested a lower DFLE for both sexes and a lower TLE among women, though a longer TLE among men, compared with current smokers. Similar differences in TLE and DFLE by smoking status were observed for groups with different levels of education.

**Conclusion:**

Never smokers live longer and healthier than current smokers and persons who quit smoking. Smoking was associated with greater reductions in TLE and DFLE among men. However, educational attainment might not moderate the adverse effect of smoking on both fatal and nonfatal conditions in the context of China. These findings have implications for disability prevention, aged care provision and informing policies of healthy aging for China and elsewhere.

## Introduction

Healthy life expectancy (HLE), measuring the expected years lived in different health statuses at a given age, is a widely used measure in gauging population health and the quality of public health systems [1–3]. According to the Global Burden of Disease Study in 2019, HLE at birth globally increased by 5.9 years from 1990 to 2015, while in China, the corresponding growth in HLE is even more significant, by 7.5 years over the same period. However, the increase in HLE in China is reported to be smaller than that in total life expectancy (TLE) (i.e., by 9.5 years over 1990–2015) [4], suggesting a greater proportion of additional years of the Chinese population to be spent in unhealthy status. This might be rooted in the growing disease burden in China due to the heightened prevalence of mild and nonfatal morbidities driven by rapid population aging.

Disability-free life expectancy (DFLE) is a frequently used measure in measuring HLE [2]. It is defined as the expected years free from physical disability or functional impairment in remaining life, representing the length of life living with independence and capacity of self-care [2, 5]. This indicator was first used in the 1970s [6] and has critical implications in reflecting the demand for aged care services [2, 7]. In the existing studies analyzing DFLE, disability is generally measured by activities of daily living (ADL), which includes six activities: self-feeding, moving, dressing, showering, using toilet and continent control [8–10].

Modifiable unhealthy lifestyle factors, which prevail in modern societies, including smoking, heavy alcohol consumption, poor eating habits (e.g., under-/over-eating and consumption of too much low-fiber and high-sugar/salt food) and physical inactivity, are important risk factors for various chronic diseases and functional impairment [11–13]. Smoking is one of the leading causes of many disabling and premature deaths [14, 15], leading to over 11 million deaths worldwide in 2015, of which over 50% occurred in China, India, the United States and Russia [16]. Previous studies have shown that smoking is associated with fewer years lived with disability due to a competing risk of mortality [15, 17–19]. For example, one study showed a four-year reduction in TLE in men and a two-year reduction in women at age 40 attributed to smoking in Japan [20]. Similar evidence from the United States reported that a nine-year loss of TLE in men and an eight-year loss in women at age 50 were due to smoking [21].

As the world’s largest tobacco consumer, China has suffered a heavy tobacco-attributable disease burden. More than one quarter of the Chinese population are current smokers [22, 23], and evidence shows that tobacco-attributable death rates and tobacco-attributable disability-adjusted life year rates have both increased significantly among Chinese men from 1990 to 2017 [22–24]. The adverse effect of smoking on health and wellbeing might vary by age, sex and educational level [25]. Specifically, the disease burden caused by smoking tends to be more severe for middle-aged and older adults due to the cumulative harmful effect caused by a long smoking experience period [19, 26, 27], and is generally more prominent among men given male higher rates in using of all tobacco products [20, 24]. Additionally, the adverse effect of smoking on health might be less significant among well-educated people given that the more socioeconomically advantaged individuals tend to smoke less and have relatively easier access to health and medical resources compared with socioeconomically disadvantaged individuals [28, 29]. However, thus far, evidence on the effect of smoking on nonfatal conditions and the role of educational attainment in this effect is apparently limited in the existing literature. In the context of China, only a few studies have focused on the role of smoking in TLE [19, 30]. This study contributes to the existing literature by investigating the impact of smoking on TLE and DFLE among middle-aged and older adults in China and also to investigate the link between smoking and DFLE by educational level based on a large nationally representative longitudinal survey.

## Methods

### Study population

This study used data from the China Health and Retirement Longitudinal Survey (CHARLS). The CHARLS is a nationally representative longitudinal database focusing on populations aged 45 and above in China. The probability-proportional-to-size (PPS) sampling technique was used in CHARLS to select respondents from all county-level units in China, covering 28 provinces, 150 counties and 450 communities [31]. The CHARLS collected detailed information on individual characteristics (such as demographic characteristics, health status and socioeconomic position) and community characteristics (such as the population and occupation constructure of communities, health facilities and socioeconomic status) by face-to-face computer-aided personal interviews (CAPIs). The baseline CHARLS survey was conducted in 2011 and was followed up every two years from 2013 to 2018. The response rates of all samples were over 80% in each wave [32].

**Fig. 1:**
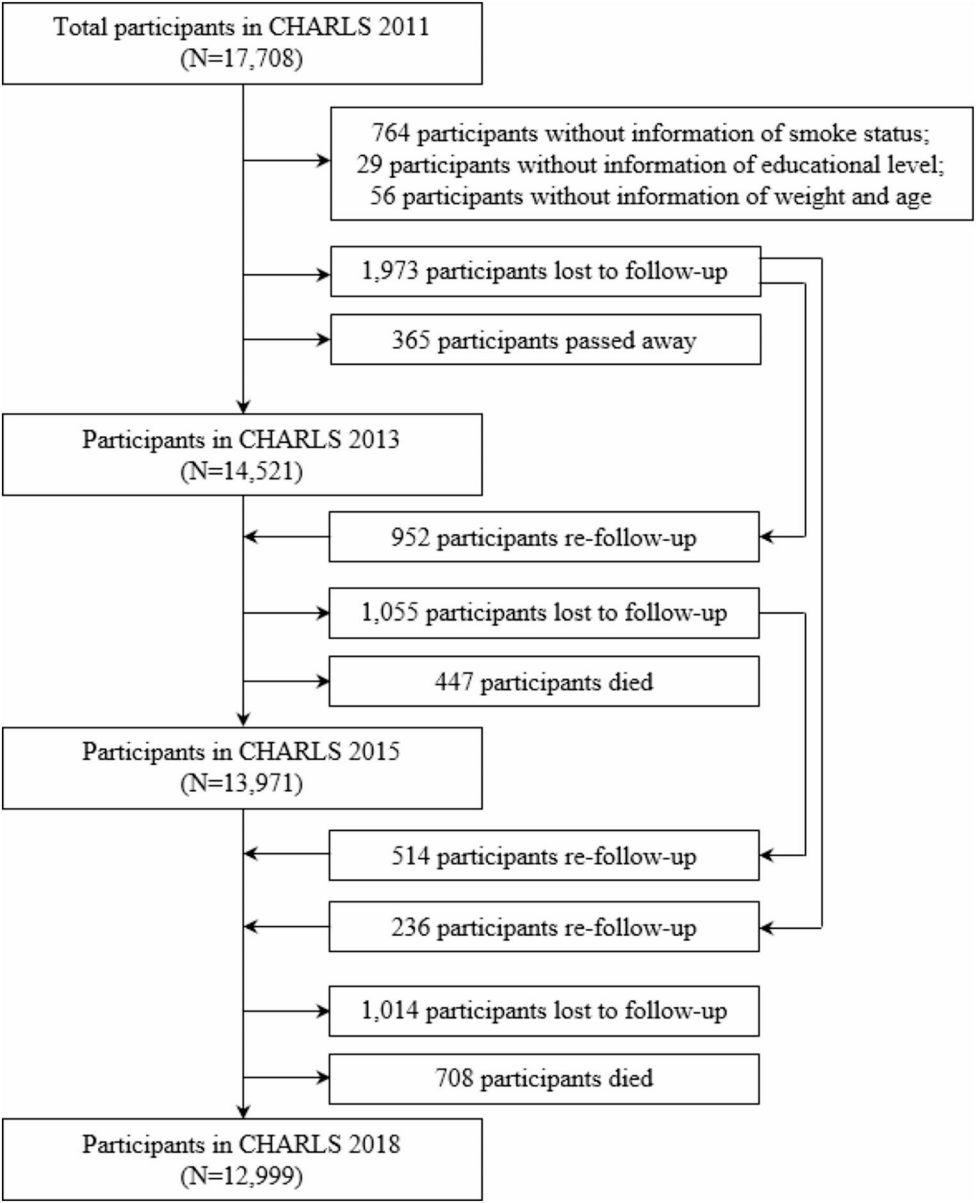
Flowchart of sample selection. *Note* although data of health status and mortality from 3,860 participants were partly missing in the four waves of CHARLS, such information can be imputed using the multistate life table method through the software Interpolated Markov Chain (IMaCh, version 0.99r8); thus, we included all 16,859 participants with their survey information over the period of 2011–2018 in the study to maintain the representativeness of the sample

This study used all four waves of CHARLS (i.e., in 2011, 2013, 2015 and 2018). Participants at baseline without information on smoking status (*N* = 764), educational attainment (*N* = 29) and age (*N* = 57) were excluded, resulting in a sample of 16,859 participants used in this study. Of the 16,859 participants, 365 died between 2011 and 2013, 447 died between 2013 and 2015 and 708 died between 2015 and 2018, while a total of 2,340 participants were lost to follow-up between 2011 and 2018, resulting in 12,999 individuals in 2018 (Fig. 1). Importantly, while data on health status and mortality from 3,860 participants were partly missing in the four waves of CHARLS, such information can be imputed using the multistate life table method through the Interpolated Markov Chain (IMaCh, version 0.99r8) software used in our study [33]. Thus, we included all 16,859 participants with their sociodemographic, health and mortality information over the period 2011–2018 in the study to maintain the representativeness of the sample (this reduced the attrition rate of the sample to 4.8%).

### Measures

#### Disability

This study used the international standard version of the ADL scale to measure disability, which consisted of six items to capture participants’ various abilities: eating, dressing, bathing, using the toilet, getting in or out of bed and controlling urination and defecation [33]. Four options were provided in response to each of the six items: (1) “No, I don’t have any difficulty”; (2) “I have difficulty but can still do it”; (3) “Yes, I have difficulty and need help”; and (4) “I cannot do it”. Participants who selected the third or fourth option of any item were considered to have a disability, while the others were considered to not have a disability. The ADL scale is a widely used tool in measuring physical function among older adults and has been demonstrated high validity [34]. In our study, the ADL scale also showed high reliability for the four waves of the CHARLS survey (Cronbach’s α = 0.86 for CHARLS 2011, 0.84 for CHARLS 2013, 0.83 for CHARLS 2015 and 0.86 for CHARLS 2018).

#### Smoking status

This study coded smoking status into three categories: current smoker, former smoker, and never smoking, which was based on responses to two questions in CHLARS: “Do you regularly smoke now?” and “Were you a regular smoker in the past?”. Participants answering “Yes” to the former question were grouped as “current smoker”, while those with an answer of “No” were categorized as “former smoker” if they answered “Yes” to the latter question and as “never smoking” if answering “No” to the latter question.

#### Educational attainment

Educational attainment was measured according to the self-reported educational level and was divided into two groups, which were “primary school and below” and “junior high school and above”.

### Statistical analysis

**Fig. 2:**
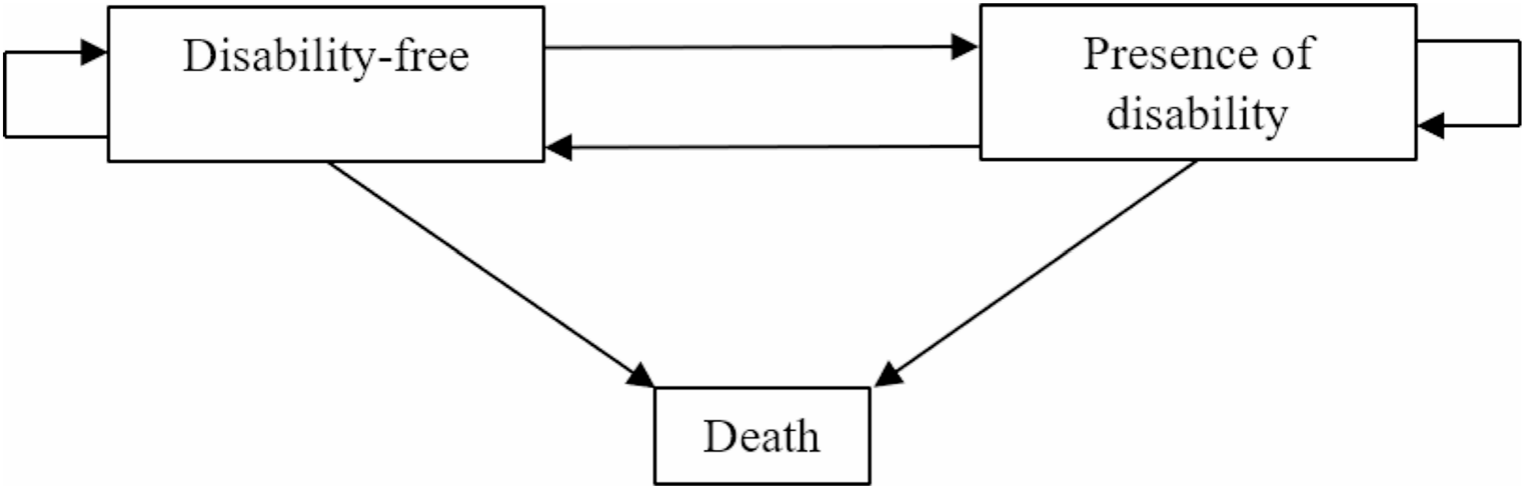
Transition pathways between disability-free status, presence of disability and death

The multistate life table method, a widely used tool in computing HLE based on longitudinal datasets, was applied in this study and was performed by the IMaCh (version 0.99r8) software. Specifically, this method computes the length of duration in different health statuses and allows a person to remain in the initial health state or to transfer to another state between two waves of a survey, a setting that has been deemed realistic and is thought to produce accurate estimation results [2]. This study included three states (disability-free, presence of disability and death) and six transitions between states (from disability-free to incident disability, remaining disability-free, from disability to disability-free, remaining disabled, from disability-free to death, and from disability to death) (see Fig. 2) to assess the association between smoking status and DFLE. Transitions between the two living states (i.e., disability-free and presence of disability) were reversible, while the transition to the death state was irreversible. All these states and transitions were calculated among participants with never, currently and formerly smoking.

The DFLE was estimated with interpolated Markov chain multistate modeling. Multinomial logistic regression was used to model the probabilities of transition from and to each living state, and the transition probability model probabilities were estimated by maximum likelihood estimations. Multistate life tables allowing for the occurrence of the transitions were constructed during this process and were constructed by sex and by educational level given the gender and educational difference in mortality and disability prevalence [33, 35]. TLE was also estimated, which was used to assess whether health expectancy estimations were consistent with mortality rates among participants. Sample weights for the longitudinal survey of CHALRS 2011–2018 were applied during the computation process. More details about the multistate modeling using IMaCh can be found elsewhere [33, 36].

The estimation results for DFLE are shown in absolute length with 95% confidence intervals (CIs) as well as in the proportions of remaining life. Stata 15.0 was also used to describe the basic characteristics of the participants.

## Results

### Characteristics of participants at baseline

As shown in Tables 1 and 28.9% of respondents out of 16,859 were identified as currently smoking, 8.5% were former smokers, and 62.6% never smoked at baseline. A total of 5.6% of participants were identified with ADL disability, and 53.8% were women, with an average age of 59.1 years old (SD = 10.1). Compared with participants with an educational level at primary school and below, those with educational attainment at junior high school and above had a lower proportion of women and disabled, a lower mean age and a higher ratio of reporting current or former smoking status. More details can be found in Table 1.

**Table 1:** Characteristics of participants in CHARLS baseline, 2011 (*N* = 16,859)

Characteristics	Total	Educational attainment	
		Primary school and below	Junior high school and above
Age, years (mean, SD)	59.1 (10.1)	61.1 (10.3)	55.0 (8.4)
Sex (n, %)			
Female	9,074 (53.8)	6,838 (60.6)	2,236 (40.2)
Male	7,785 (46.2)	4,455 (39.4)	3,330 (59.8)
Smoking status (n, %)			
Never	10,554 (62.6)	7,396 (65.5)	3,158 (56.7)
Current	4,867 (28.9)	2,985 (26.4)	1,882 (33.8)
Former	1,438 (8.5)	912 (8.1)	526 (9.5)
Disability (n, %)			
Yes	947 (5.6)	779 (6.9)	168 (3.0)
No	15,912 (94.4)	10,514 (93.1)	5,398 (97.0)

### Associations of smoking status with TLE and DFLE

Tables 2 and 3 demonstrate the relationship between smoking status and TLE and DFLE by educational attainment in men and women. For both men and women aged 45 years old, TLE and DFLE were lower among both current and former smokers compared with never smokers (men: TLE loss = 2.4 for current smokers and 2.8 for former smokers, DFLE loss = 1.7 for current smokers and 2.6 for former smokers; women: TLE loss = 1.5 for current smokers and 1.0 for former smokers, DFLE loss = 0.8 for current smokers and 1.5 for former smokers). Additionally, the magnitudes of the reduction in years of TLE and DFLE were both prominently larger in men than in women.

**Table 2:** Total life expectancy (TLE) and disability-free life expectancy (DFLE) by smoking status and educational attainment, with 95% confidence interval, men

		Total	Primary school and below	Junior high school and above
		**TLE**		
Age 45	Never smoking	36.0 (35.4–36.5)	35.1 (34.5–35.6)	37.8 (37.1–38.5)
	Current smoking	33.6 (33.2–34.0)	32.9 (32.5–33.3)	35.5 (34.9–36.2)
	Former smoking	33.2 (32.6–33.8)	32.3 (31.6–33.0)	35.0 (34.2–35.8)
Gain (+) /loss (-) ^a^	Never smoking	Reference	Reference	Reference
	Current smoking	-2.4	-2.2	-2.3
	Former smoking	-2.8	-2.8	-2.8
Age 65	Never smoking	18.3 (17.9–18.7)	17.7 (17.2–18.1)	20.0 (19.4–20.6)
	Current smoking	16.4 (16.1–16.7)	15.9 (15.6–16.3)	18.1 (17.6–18.6)
	Former smoking	16.3 (15.8–16.8)	15.7 (15.2–16.2)	17.9 (17.2–18.5)
Gain (+) /loss (-)^a^	Never smoking	Reference	Reference	Reference
	Current smoking	-1.9	-1.8	-1.9
	Former smoking	-2.0	-2.0	-2.1
		**DFLE**		
Age 45	Never smoking	32.7 (32.3–33.2)	31.8 (31.3–32.3)	34.7 (34.1–35.3)
	Current smoking	31.0 (30.7–31.4)	30.2 (29.8–30.6)	33.1 (32.6–33.7)
	Former smoking	30.1 (29.6–30.7)	29.1 (28.5–29.7)	32.0 (31.3–32.7)
Gain (+) /loss (-) ^a^	Never smoking	Reference	Reference	Reference
	Current smoking	-1.7	-1.6	-1.6
	Former smoking	-2.6	-2.7	-2.7
Age 65	Never smoking	15.3 (15.0-15.7)	14.7 (14.3–15.0)	17.1 (16.6–17.6)
	Current smoking	14.1 (13.8–14.3)	13.6 (13.3–13.9)	15.9 (15.4–16.3)
	Former smoking	13.4 (13.0-13.8)	12.8 (12.4–13.2)	15.0 (14.5–15.6)
Gain (+) /loss (-)^a^	Never smoking	Reference	Reference	Reference
	Current smoking	-1.2	-1.1	-1.2
	Former smoking	-1.9	-1.9	-2.1

Tables 2 and 3 also show that former smokers had a lower figure than current smokers in DFLE at age 45 for both sexes (30.1 years [95% CI: 29.6–30.7] vs. 31.0 [95% CI: 30.7–31.4]) for men, 31.9 [95% CI: 31.2–32.5] vs. 32.6 [95% CI: 32.1–33.0] for women) and a lower TLE at the same age among men (33.2 [95% CI: 32.6–33.8] vs. 33.6 [95% CI: 33.2–34.0]). However, former smokers had a longer TLE at that age (37.0 [95% CI: 36.2–37.6] vs. 36.5 [95% CI: 35.9–37.1]) than current smokers among women. Similar patterns were found at age 65.

**Table 3:** Total life expectancy (TLE) and disability-free life expectancy (DFLE) by smoking status and educational attainment, with 95% confidence interval, women

		Total	Primary school and below	Junior high school and above
		**TLE**		
Age 45	Never smoking	38.0 (37.7–38.4)	37.7 (37.4–38.0)	40.6 (39.9–41.3)
	Current smoking	36.5 (35.9–37.1)	36.4 (35.8–37.0)	39.2 (38.4–40.0)
	Former smoking	37.0 (36.2–37.6)	36.5 (35.7–37.3)	39.5 (38.5–40.5)
Gain (+) /loss (-) ^a^	Never smoking	Reference	Reference	Reference
	Current smoking	-1.5	-1.3	-1.4
	Former smoking	-1.0	-1.2	-1.1
Age 65	Never smoking	19.9 (19.6–20.2)	19.7 (19.4–20.0)	22.3 (21.6–22.9)
	Current smoking	18.5 (18.0–19.0)	18.5 (18.0–19.0)	21.0 (20.2–21.7)
	Former smoking	19.2 (18.5–19.8)	18.9 (18.2–19.6)	21.5 (20.6–22.4)
Gain (+) /loss (-)^a^	Never smoking	Reference	Reference	Reference
	Current smoking	-1.4	-1.2	-1.3
	Former smoking	-0.7	-0.8	-0.8
		**DFLE**		
Age 45	Never smoking	33.4 (33.1–33.6)	33.0 (32.7–33.2)	36.1 (35.6–36.7)
	Current smoking	32.6 (32.1–33.0)	32.4 (31.8–32.9)	35.6 (34.8–36.3)
	Former smoking	31.9 (31.2–32.5)	31.3 (30.6–32.0)	34.4 (33.6–35.2)
Gain (+) /loss (-) ^a^	Never smoking	Reference	Reference	Reference
	Current smoking	-0.8	-0.6	-0.5
	Former smoking	-1.5	-1.7	-1.7
Age 65	Never smoking	15.6 (15.4–15.9)	15.4 (15.2–15.7)	18.1 (17.6–18.6)
	Current smoking	15.0 (14.6–15.5)	15.0 (14.5–15.4)	17.6 (17.0-18.3)
	Former smoking	14.5 (14.0-15.1)	14.2 (13.6–14.8)	16.7 (16.0-17.4)
Gain (+) /loss (-)^a^	Never smoking	Reference	Reference	Reference
	Current smoking	-0.6	-0.4	-0.5
	Former smoking	-1.1	-1.2	-1.4

Tables 2 and 3 further show that the effect of smoking on TLE and DFLE was largely similar for each educational level. For example, compared with the similarly educated male never-smokers, male current smokers with an educational level at primary school and below saw a reduction of 2.2 years in TLE at age 45; this was very close to the gap of 2.3 years in TLE at that age between male current smokers with an educational level at junior high school and above and male never-smokers in the same educational level. Likewise, compared with similarly educated female never-smokers, female former smokers with an educational level at primary school and below experienced a 2.7-year reduction in DFLE at age 45, which was exactly the same difference in DFLE at that age between female current smokers with an educational level at junior high school and above and female never-smokers with the same educational level.

**Fig. 3:**
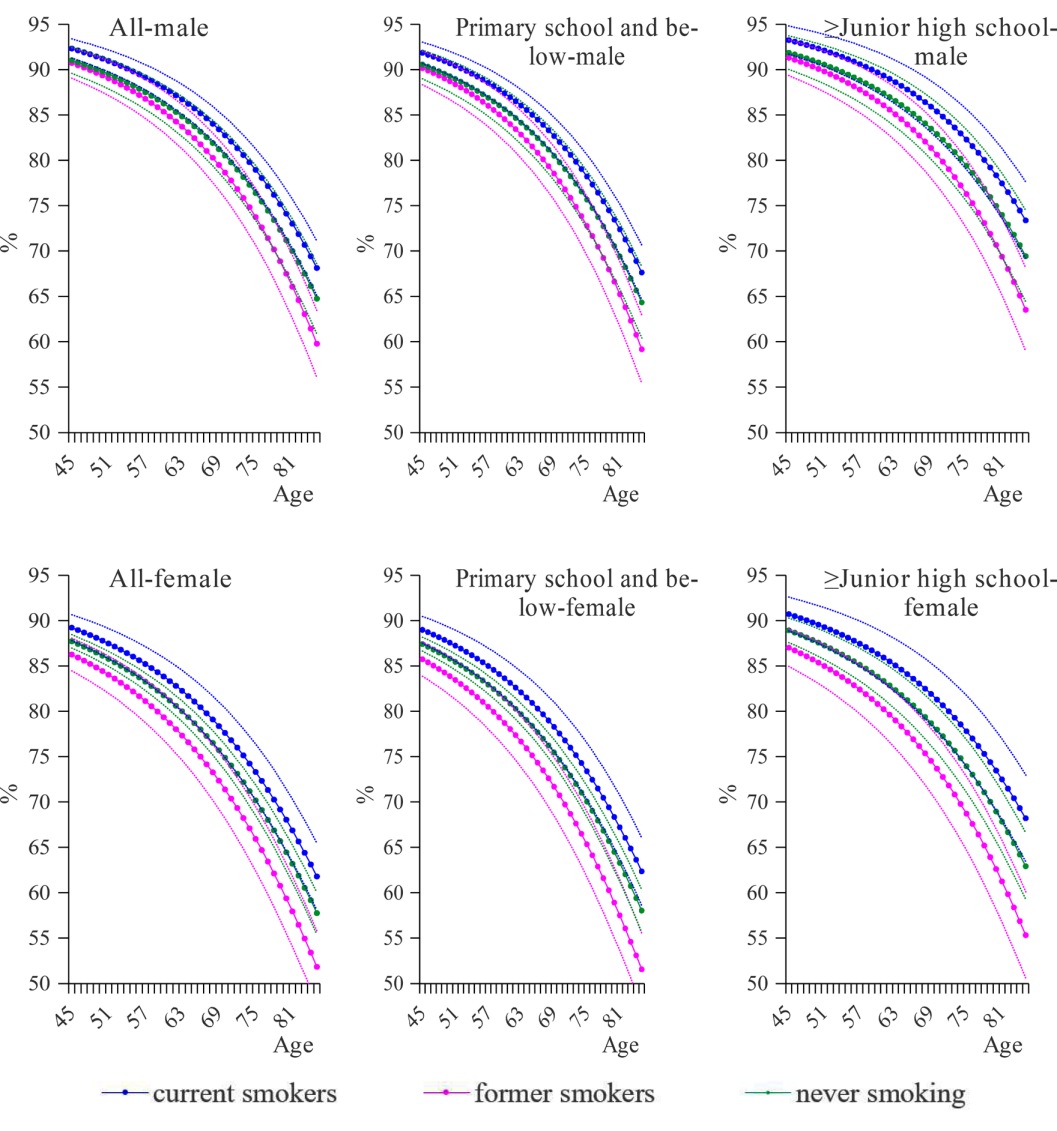
The proportions of DFLE in the remaining life for participants by smoking status and educational attainment. *Note* dotted lines denote 95% confidence intervals

Figure 3 presents the proportions of DFLE in the remaining life for participants by smoking status and educational attainment. Clearly, for both women and men, former smokers had the lowest proportions of DFLE in TLE. Although there were fewer years of DFLE and TLE for current smokers than for never smokers, current smokers had the highest proportion of DFLE in the remaining life. Similar variations were found in groups by educational attainment.

## Discussion

This study is the first to examine the effect of smoking on DFLE by using a nationally representative longitudinal survey in the context of China. Smoking was found to reduce TLE and DFLE regardless of sex and educational attainment among middle-aged and older adults in China, demonstrating that middle-aged and older adults suffer a high risk of mortality and morbidity from smoking. Furthermore, this study also found that smoking was associated with larger differences in TLE and DFLE in men than in women, suggesting a disproportionally high smoking-related disease burden among middle-aged and older men. However, we did not observe significant differences in TLE and DFLE by educational level, as expected.

The reductions in TLE and DFLE attributed to smoking have been reported in previous studies. Loss in years of TLE at age 50 among men and women due to smoking was found to be 8.66 years in men and 7.59 years in women in the Framingham Heart Study [21], while reduction in DFLE at age 50 due to smoking was found to be 3.5 years and 3.0 years among men and women, respectively, in four European countries [37]. This study responds to previous findings, demonstrating that smoking exerts a significant shortening effect on both TLE and DFLE and that the loss of years caused by smoking are largely disability-free years. This implies a strong adverse effect of smoking on length and, more importantly, quality of life among middled-aged and older adults. The detrimental effect of smoking on health in middle and later life should raise increased concerns from policymakers and care providers, particularly those in China, where people aged 45–64 have a higher prevalence and a greater intensity of smoking compared with other age groups [25, 38], and the population size of this age group is expected to grow remarkably in the coming decades given rapid population aging [39].

Our study also suggests that smoking was associated with a greater reduction in TLE and DFLE in men, with men losing 2.4 years in TLE and 1.7 years in DFLE for current smoking compared with 1.7 and 0.8 years in women, which may be because men generally smoke much heavier than women. In China, the prevalence of smoking among people aged 15 and older reached 52.9% among men and only 2.4% among women in 2010 [25, 38]. In addition, the prevalence of smoking among Chinese men is even higher than that of their male counterparts in some developed countries, such as the United States (21.5% for men aged ≥ 18 in 2010) [40] and the United Kingdom (19.3% for men aged ≥ 18 in 2015) [41]. The sharp gender difference in the prevalence of smoking in China and the higher proportion of Chinese men currently smoking than many other countries suggest that Chinese men are exposed to a disproportionally severe detrimental effect of smoking on their health, which is hence reflected by the substantial loss in TLE and DFLE observed in this study. This finding warrants greater efforts for the anti-tobacco campaign and smoking cessation interventions among the male population, particularly in countries such as China, where men exhibit a high smoking rate, but the sociocultural context has been challenging for them to quit smoking (e.g., Chinese cigarette gifting customs) [42].

Our findings also show that former smokers experienced a greater reduction in DFLE than current smokers among both sexes and a greater TLE reduction among men (all using never-smokers as a reference). This intriguing finding may indicate that the cumulative harmful effect may not be fully eradicable even after smoking cessation [43]. More specifically, while most of the smoking-caused changes can be reversed after smoking cessation, some of the adverse consequences of smoking, such as inflammatory mediators, still occur among ex-smokers even after up to 20 years, suggesting a longer-term effect of smoking even after quitting, which might reduce former smokers’ quality of life [44]. The greater loss in DFLE in former smokers compared with current smokers might also be explained through their quitting reasons. One study reported that the most common reason triggering smoking cessation in China is illness (approximately 30%), compared with approximately 12% due to education. This comparison becomes even sharper among people aged 45 and older (43% vs. 5.9%) [45]. This suggests that people in middle or old age might be already ailing when quitting smoking and hence exhibit a relatively low DFLE observed in this study.

Furthermore, this study did not detect significant differences in terms of the effect of smoking on TLE and DFLE by educational level. This is opposite to the general understanding of a positive association between education and health outcomes; however, this might be explained by the unique smoking pattern by educational level in the context of China, which sees a generally lower prevalence of smoking among those ill-educated compared with those better-educated. A national population-based survey in 2010 reported that among people aged 15 and older, the smoking prevalence for those with an educational level at primary school or less numbered at 24.6%, significantly lower than that of those with an education level up to secondary school at 36.1% and those with an education level up to high school at 34.9%, but was similar to those with a bachelor degree and above at 26.6% [25]. In our study, the proportions of current smokers and former smokers were also both lower among participants with an educational level at primary school and below (26.4% and 8.1%, respectively) than among those with an educational level at junior high school and above (33.8% and 9.5%, respectively). The relatively higher smoking prevalence among well-educated participants in China might offset the benefits brought by education and hence result in the similar magnitudes in terms of smoking’s effect on TLE and DFLE by educational level detected in this study.

In addition to the previous discussion, it is noteworthy to recognize the relatively low smoking rates among women in China compared to men (1.62% vs. 44.52% in 2018) [46]. This situation presents a fortunate circumstance for China, as smoking-related health issues can have far-reaching consequences for individuals and society at large. To preserve this clear advantage, it would be wise for China to proactively establish a comprehensive smoking prevention program specifically targeting women to contain the gradually increasing smoking rate among Chinese female population [47]. By focusing on education, awareness campaigns, support services and other tailed intervention measures considering the gender differences in smoking cessation, such a program could effectively empower women to resist the allure of smoking and maintain their lower smoking rates. By prioritizing provision of better aligned cessation care for women in smoking quitting campaign, China can further fortify its progress in reducing smoking prevalence and ensure a healthier future for all its citizens.

## Limitations

To the best of our knowledge, this study is the first to examine the association of smoking with DFLE among middle-aged and older adults in the context of China by using a longitudinal national representative survey. The results highlighted the nonfatal health consequences of smoking among Chinese middle-aged and older adults. Given that CHARLS is part of a larger set of surveys (e.g., the Health and Retirement Study in the United States, the English Longitudinal Study of Ageing in England, the Korean Longitudinal Study of Ageing in South Korea and the Mexican Health and Aging Study in Mexico), our findings would allow later comparisons between countries in the future. However, some limitations still existed in this study. First, due to the restriction of our dataset, the duration and intensity of smoking were not clearly examined in our analysis, which may lead to bias in the estimations. Second, this study did not include institution-dwelling persons in institutions but only involved community-dwelling older adults. Thus, our estimations of life years may be overestimated due to the omission of older adults in institutions that have worse health status. Third, due to the inevitable missing data in the large national longitudinal study, caution should be taken in interpreting the results. Fourth, while this study provides the first investigation into the differences of TLE and DFLE attributable to cigarette smoking and the related variations by gender and educational level, it has not examined such variations from other dimensions, such as comorbid conditions, income level, marital status and various lifestyle factors. This is because if we included all the three dimensions (age, gender, and educational attainment) and other variables (e.g., comorbid conditions and various lifestyle factors) at the same time, the computation might not process successfully. With each additional variable in the computation process of the multistate life table method, the probability for a successful convergence of the matrix decreases, which might lead to a failure in producing final results. We have not adjusted income level because a significant amount of missing data regarding the income variable, at approximately 40%, were missing in the CHARLS dataset. Future research is recommended to explore more dimensions based on other reliable data sources, and use computation methods other than the multistate life table method to validate our findings.

## Conclusions

This study showed that smoking was associated with reductions in both TLE and DFLE among middle-aged and older adults in China. Smoking was associated with larger differences in TLE and DFLE in men than in women, while the TLE-/DFLE-shortening effect of smoking was similar by educational level in the context of China. These results are useful in health management, disability prevention and intervention and in informing health policies for policy makers in China and elsewhere.

## Data Availability

The datasets generated during the current study are available in http://charls.pku.edu.cn/.
